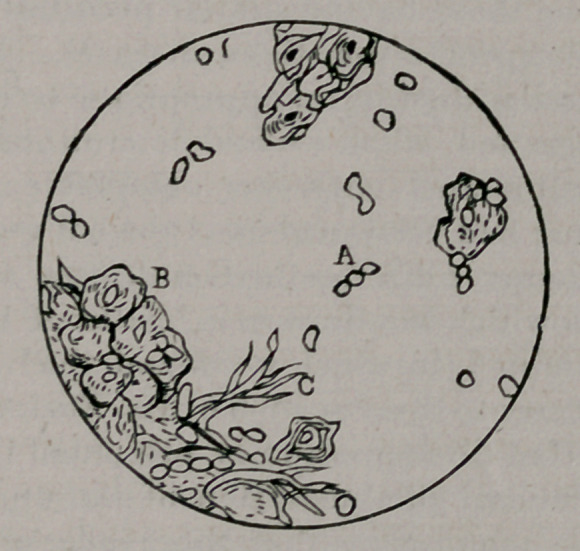# Impetigo Contagiosa

**Published:** 1886-08

**Authors:** E. J. Beall

**Affiliations:** Fort Worth, Texas


DANIEL'S

### Medical ★ Journal,

PUBLISHED MONTHLY AT

AUSTIN, TEXAS.

Vol. 2.] AUGUST, 1886. [No. 2.

"*Scribimus indocti, doctique!*"

### Original Articles.

CONTRIBUTED EXCLUSIVELY TO THIS JOURNAL.

*The Articles in this Department are accepted, and published with the understanding that we are not responsible for, nor do we indorse the views and opinions of the writers, by so doing.*

### IMPETICO CONTAGIOSA.

*By E. J. Beall, M. D., Fort Worth, Texas.*

(For Daniel's Texas Medical Journal.)

TO Tilbury Fox, twenty-two years ago, are we indebted for the first description of the disease, the title of which heads this article. Since the date mentioned, many writers on dermatology have referred to the affection; not lengthily, however, as no one writer upon diseases of the skin seems to have enjoyed very many opportunities for its observation.

While in the main most dermatologists fairly agree in description, etc., yet some discrepancies do exist, and upon points of importance as bearing upon a correct portraiture, ætiology, etc. It is with less disposition to make an effort to settle any existing conflictions (of whatsoever character, as to variations or discrepancies), than the desire to bring the subject here presented to the notice of the general practitioner; for peradventure, at any moment the disease may occur in his daily work, and a need would then arise for its proper recognition, as the predicate for a correct therapy.

Twice, in thirty years, have I recognized in practice contagious impetigo; about five years ago, having observed ten or twelve cases, and quite recently twelve others. What I shall here write is drawn from observations made upon the cases last mentioned, as they are fresh in mind, and consequently deductions would be of greater value.

The first case was a child six years old. From this originated mediately or immediately the following:

In one family, six children, whose ages varied from 15 months to 19 years, and the *pater-familias*, aged 52 years; in four other families five cases, the patients aged 4 to 7 years. All of these children were playmates, and closely associated with one another.

It will be observed that the youngest subject was 15 months old; the oldest 52 years. In only five of the cases was there appreciable fever, and that of only two days duration. In the boy, aged 19, much pain was complained of in the lumbar region, and pulse was reduced to fifty per minute. Whether this circulatory phenomenon was a consequence, or coincidence, I am not prepared to say.

The eruption appeared upon the face in only seven cases. On each of two cases, only four "vesico-pustules" were seen; these at either anterior axillary border; in shape resembling a figure 8, and as large as two silver half dollar pieces; edges approximated. The largest "vesico-pustule" was upon the oldest subject (the only one on him), and was located upon the inner aspect of the calf of the leg.

All the cases occurred in the healthiest and most fashionable portion of the city of Fort Worth; in families of average health in the community, who have always been well fed and cared for.

What I shall present in this paper is based upon a personal observation of the cases whose ages and environment are herein given; and not drawn from descriptions already before the profession, notwithstanding the subject has received attention from the oldest dermatologists. I write only of that which I saw, or rather, as the observation of the disease impressed me. Whosoever is sufficiently interested, can run the parallels and reconcile discrepancies of observation between myself and others; and I will be excused in remarking that this method is the keynote in clinical medicine, and brings afresh to mind the oft' quoted extract from Morgagni:

"Nulla est alia pro certo noscendi via, nisi quam plurimas et morborum, et dissectionum historias, tam aliorum proprias, collectas habere et inter se comparare."

*Definition*—Contagious impetigo is a semi-acute, slightly inflammatory, contagious disease, showing one to twenty, or more, very superficial, discreet or confluent "vesico-pustules" which rapidly form thin, yellow or amber-colored, easily detachable crusts, upon removal of which a moist, excoriated surface is presented. A secondary crust (maybe traumatic) often follows the removal of the primary one. The central detachment of either is followed by a pale red or violaceous surface, which passes to a copper-red or murky brown, the discoloration sometimes remaining two or three or more weeks. The maculation is frequently distributed so as to present the appearance of a ring within a ring—occasioned, most probably, by the crust detachment taking place at different periods.

### SYMPTOMATOLOGY AND DIFFERENTIAL DIAGNOSIS.

Impetigo contagiosa may or may not begin with constitutional symptoms of a pyrexial character. At first, vesicles with, or without, slight peripherical* redness, or areolæ, are seen. These vary in size from that of a half pea, or larger, and may rapidly enlarge until, in some instances the dimension of a silver half dollar is attained. The process of enlargement occurs in a few hours, or within the course of a day. The semi-transparent fluid of the primary vesicle gradually changes color, becoming more opalescent—the vesicle-capsule as well as its contents undergoing the same transition. This

NOTE.—I make a discrimination between the terms "peripheral" and "peripherical." See Thomas' Medical Dictionary.

change from partial transparence to opalescence will be considered further on.

The vesico-blebs, or "vesico-pustules," if few in number, and located upon the trunk or limbs, may escape the notice of both patient and physician, as in the majority of cases there is neither irritation nor itching. The vesicles are usually terminated by maturation or an imperceptible traumatism. There is no regularity in their number, size and location upon the body—they may be few, small, medium or large, and occupying diverse situations. When two or three days have elapsed, the vesico-blebs, or "vesico-pustules," break, either from maturation or traumatism. The cuticle is now observed to shrink; to collapse; to be too small to cover the original area of the vesico-bleb, or "pustule," while its surface is marked with parallel linear wrinkles.

The vesico-bleb, or "pustule-capsule," is pertinently mentioned; as *all writers* note this physical condition, but do not refer to the occurring superficial histological changes, through which a pathological conclusion may be arrived at, upon which might be based correct or deductive propositions in diagnosis.

When the vesico- blebs have broken, their contents poured out, leaving a somewhat excoriated surface, then ensues the primary crust formation. These primary crusts, or scabs, resemble brown sugar or cera flava in color, are flaky, have a precarious hold to the surface—as if "stuck on"—and when matured are easily detached by thumping, or by slight nail interference. When crust detachment has occurred, the peculiar primary maculated surface is observed to be slightly red or violaceous in color, and perceptibly moist. When the primary crust is removed, especially should the removal be premature, there may take place a secondary crust. This is not, however, of constant occurrence; yet 'tis not infrequent. A solution of the secondary crust formation may be that, it is resultant upon the interference with the natural course of maturation of the primary crust. Yet, other hypotheses might be presented as solutions of the fact of their formation or existence.

It will be quite frequently observed, in relation to the crust detachment, that separation occurs at the center first, progresses centrifugally, so that after a day or two only a narrow peripheral attachment remains which, in turn, soon disunites. This cond tion I generally observed in the cutaneous lesions covered by clothing. The delayed peripheral detachment of the crust does not exist in every case, nor in each incrustation of a given case; but is more particularly noticeable in connection with the larger incrustations.

I have heretofore referred to a consequence of this peculiar detachment, in the maculation of the site preoccupied by the crust; showing a variation of maculation, in concentric rings, darkest at the periphery, growing lighter centripetally. I have positive evidence of this maculation remaining three months or more after the crust is removed.

I have also called attention to the sites selected by the disease as they occurred under my observation. As stated, in no instance have I seen a mucous membrane involved. Duhring and other dermatologists have noted this disease as attacking that membrane.

It is stated that the disease will continue from six to fifteen days. I think that, waving exceptions to be noted, from incubation fever to vesicle formation is two or three days, and from vesicle formation to falling of crust six or eight days. There are, perhaps, abortive cases; but my observations have been too limited to make this a positive assertion. As to chronicity: When a child, or an adult, particularly the former, is once infected, the termination of the disease becomes a matter of environment, location of lesions, and treatment instituted. When the cutaneous lesions are upon the face or chin, particularly of children, my observations point to the greater liability of the disease being protracted. The pathogenetic effect is here protracted by scra ching, want of cleanliness (in children), and auto-infection ; but even under such circumstances I have seen one or two cases only in which the disease continued longer than four or five weeks--waving the maculation which follows the com| lete detachment of the crust, and complete dessication of the part occupied by the disease.

Again, perhaps the increased itching when the disease occupied the face, chin, etc , favored, as has been hinted at, a more chronic course. In such cases, the neck, particularly the anterior cervical creases, showed the evidences of the disease in marked degree; but here the lesions were smaller, much smaller than the original incrustations, whence, perhaps, the fungi had been transferred.

In speaking of the peripheral maculation being of deeper shade than at the center, and in referring to the fact that at the periphery there was now and then observed the falling crust a day or so after the center was clear, it might be interesting to account for this condition in a hypothetical way.

*A.* Does the detachment begin at the center because the disease began there; and attachment of the crust hold at the periphery later because the disease left the circumference last?

*B.* Do the fungi or micrococci begin their life-work at the center and consume that which nature elects for their use, and working from the center to the circumference end their life-work, nature, in the meantime, repairing the habitation of their first election ere they have vacated their last?

*C.* Do they have a limited life-tenure of ten or fifteen days, and when from a given point to a circumference they have consumed that infra-dermic pabulum suited to their growth and maturation, then ensues a peripheral gathering, and by their death or ptomaines leave the last evidence of their existence in deeper peripheral maculation, and delayed falling of the peripheral portion of the crust?

### ÆTIOLOGY AND PATHOLOGY.

Dermatologists differ as to the cause of impetigo contagiosa. Some assume the existence of a fungus as the prime *materies morbi*, while others claim that no fungus can be demonstrated; and others still, contend there is a relation existing between the disease and that of vaccination, and that the fungus, or parasite, is identical with that found in the vaccine crust. That it does sometimes follow vaccination, I am prepared to acknowledge and believe; for when I observed my first cases, a few years since, smallpox was prevalent, and very general vaccination had been and was being done. The latter cases, those upon which the observations herein given were made, I know did not follow immediate vaccination. In a portion of the twelve cases lately observed, not one had been vaccinated nearer to the development of the disease than four months; and in several of those affected with impetigo contagiosa, who were vaccinated four months prior to the appearance of the disease, no effect could be induced by vaccination, because protected by previous and repeated vaccination in the years preceding.

In the study of the microscopy of the crust removed from one of the twelve cases, it would have been agreeable, and perhaps instructive, to have made a parallel study of a vaccine crust, and to have endeavored to negative or affirm the work of Dr. Piffard in that line; but a vaccin crust was not attainable. I have stated that a number of the cases lately observed had been repeatedly vaccinated not, however, within four months of the time when contagious impetigo was contracted. If the fungus is, as has been claimed, the same, it would be interesting to know whether one having had contagious impetigo is protected against smallpox. If not, a paradox is presented that needs the touch of time and the hand of a master to elucidate. Of one thing, however, in this dilemma, I am certain: Vaccination is *no* protection against impetigo contagiosa! And another; contagious impetigo does not respect previous smallpox, as one of the subjects of that disease was the subject of variola a number of years ago.

Believing the genesis of the disease is dependent upon a peculiar micro-organism, the evidence upon which this belief is based is here presented for the consideration of the reader:

*Microscopical Examination of the Primary Crust from Impetigo Contagiosa.*

The crust was treated with a three per cent. solution of pure caustic potash, then stained with eosin, and mounted in glycerine, upon a glass slide.

Observations were made with a one-fifth Gundlach objective, D eye-piece and amplifying tube.

By staining, a happy result was obtained—perfect differentiation became easy. The field presented an abundance of epithelium and fibrous tissue; here and there oil-globules and crystals of margarine; and, scattered throughout, numerous minute, round and oval bodies which were considered to be the micro-organism constantly attending, and in all probability the direct factor in the ætiology of the disease (*impetigo contagiosa*).

In the method used, the epithelium and the elastic fibres partook of the stain, while the micro-organisms did not. The nuclei of the epithelial cells were well defined; and the elastic fibres, owing to their refractive property, were easily discerned. The oil-globules presented only such features as are common to them when viewed in glycerine menstrua; a few, however, were contaminated with detrital granules, in which instance their appearance resembled bi-concave disks and nucleated cells The crystals of margarine were identical with that found in sebaceous matter, perhaps somewhat more highly colored. The micro-organisms, under white light, were colorless, highly refractive, spheroidal and ovoidal in shape; $\frac{1}{8000}$ to $\frac{1}{5000}$ of an inch in diameter; disseminated throughout the field, singly and in pairs, sometimes, *apparently*, in chaplets of three and four. In a few instances they *appeared* to be moniliform, but further investigation disproved that condition, the appearance having originated in the juxtaposition of two or more pairs. From the many observations made, the following data were obtained:

In early life the organism is an exceedingly small, transparent spherule, not exceeding $\frac{1}{8000}$ of an inch in diameter (some of them measured as low as $\frac{1}{10000}$ of an inch in diameter); highly refractive; not acted upon by alkalies, nor capable of being stained by eosin. From the spherule they pass to an ovoid, which, evidently, is their form at maturity. The diameter measurement of the ovoidal forms exhibited a variation from $\frac{1}{8000}$ to $\frac{1}{5000}$ of an inch; and in no instance could there be detected an ovoid of less diameter than the smaller figure noted, or of greater than the larger. Propagation proceeds by cell division, or "segmentation." When the organism reaches maturity, unless it dies, a nebulous line crossing it transversely can be readily detected. If this line is carefully watched, it will be seen to deepen to a furrow which completely encircles the ovoid at right angles with its length. The constriction steadily progresses until the original ovoid assumes the appearance of two attached spherules, which, in time, should segmentation become complete, separate, each beginning an individuality. If these newly formed individuals live, and their life career is observed, they will be seen to undergo the same transformation witnessed of their progenitors: from the spherule to the ovoid, then segmentation. It frequently happens that segmentation does not reach maturation. In such instances the organism becomes a dyad; but if it remains in this condition it is most likely due to the advent of death—had life continued, the separation would have been complete. Where more than two spherules or ovoids appear to be connected, the multiplicity is owing to juxtaposition of individuals. As hereinbefore stated, the micro-organisms found in the crusts from impetigo contagiosa are not of the moniliform variety—they do not exist in chaplets. Nor are they hyphomycetous, as neither *hyphæ* nor *mycelia* were present. The number of organisms invading the epithelium and elastic fibres were in direct ratio with the square of the surface exposed, each tissue contributing its proportion.

The accompanying illustration is rather a poor representation of the view it is intended to reproduce. It is to be regretted the engraver was not more painstaking; as it is, great injustice has been done the observation. Delicate and accurate definition has been sacrificed, and only a crude sketch remains.

A is intended to represent the juxtaposition of an ovoidal micro-organism to a pair (dyad) in which segmentation was nearly complete. That the ovoid was not connected with the "dyad" could be readily and positively determined in the original; but not so easily in the illustration. B is a mass of epithelial cells, many of which were invaded by the organism. The engraver failed to differentiate between the nuclei of the epithelial cells and the organisms. C, elastic fibres.

While the disease is considered to be extremely superficial, it evidently penetrates the epidermis and papilla, and involves, to a limited extent, the superficial layer of the corium. The organisms were found in, and the crusts contained the exuded elements of, the tissues mentioned.

In attempting to classify this organism, many difficulties are encountered; but in the light of our present knowledge it may be safely said to be a protophite belonging to the order of schizomycetes; suborder, sphærobacteria; genus, micrococcus.

If further investigation should determine that the microbe of contagious impetigo is in fact a micrococcus, reasoning by analogy, when studying its symptomatology, etc., we may conclude that the disease must be classed with the infectious diseases, as varicella, etc. Several writers say the disease sometimes prevails epidemically. There is sometimes a fever stage which precedes the skin manifestation; also, a regularity in the progression of the eruption. These and other facts might be noted as indices for the propriety of classifying the affection under consideration among those having similar peculiarities; and such obtains with the specific infectious diseases. Would not *impetiginoid varicella*, then be an appropriate term?

It is to be regretted, when we had determined to investigate this disease, that a crust only was obtainable; that not until after the drawing had been made and the cut received from the hands of the engraver did the thirteenth case come under observation, from which the fluid of a matured vesicle was obtained and examined, in which we found the identical micro-organism (contrary to the assertion of Dr. Fox) that was present in the crust. Had this opportunity presented itself earlier, we would have produced an illustration of the appearance of the fluid under the microscope that the reader might make the comparison with that of crust.

It would have been a matter of further interest to have subjected the microbes to culture, and endeavored to propagate the disease from attenuated cultures of both fluid and crusts. But it otherwise happened, and the consolation only remains that, the future may yet bring with it the opportunity of inves-tigating this and other points of paramount interest.

Let us commend the broad and interesting field referred to to others; and, although the disease does not endanger life, there is a point, peradventure involving human happiness or woe, which imperatively demands of our profession as nearly perfect elucidation as practicable that a differentiation, from specific disease, at least, may be established. He who shall work this field well, we think, may establish an oasis in a desert; may place a hand-print upon medical work and literature.

DIAGNOSIS.

The disease is to be differentiated from varicella, eczema pustulosum, impetigo, ecthyma, syphilis, etc.

*From Varicella.*—The vesicles and vesico-pustules of varicella are, as a rule, more generally disseminated over the body; are smaller; and differ in consistence and color from that which obtains in impetigo contagiosa.

*From Eczema Pustulosum.*—In the latter disease there is no fever, the surface is redder, crusts thicker and darker and not so easily detached; while the itching, discharge and cutaneous infiltration are greater.

*From Impetigo* —Impetigo is always pustular, or primarily so; is more apt to appear in patches; the pustules are smaller and deeper seated.

*From Ecthyma; Simple or Specific.*—In this disease the eruption is discrete, pustular, and situated upon an inflammatory base (notably so as regards non-specific ecthyma); forms thick dark scabs, and is painful (especially in the non-specific variety); is also of longer duration, as a rule.

*From the Pustular Syphiloderm* —In this case the lesions are usually small, and grouped; crusts more adherent; are "thick, bulky, uneven and heaped-up;" maybe greenish-yellow or dark; the ulcer beneath, deeper.

In making a differentiation from the *disease* just mentioned, or *others*, I wish to *emphasize* the following:

In very many cases of contagious impetigo when the crusts detach, they begin at the center and progress towards the circumference; and when the first shedding is complete, the center will be clear, while at the periphery a thin incrustation remains. One or two days later this thin incrustation exfoliates. Again, I wish to emphasize the fact that, the maculation which follows the crust detachment is lightest in color at the center, where separation first began, and darkest at the periphery, where exfoliation last occurred. This condition is not always seen; but I observed it in a number of cases, in which it was generally observed upon the neck or at unexposed places. I desire to make it a matter of record that, when seen, it can be *considered pathognomonic of the disease.*

PROGNOSIS.

May be considered always favorable. If an unfavorable termination should occur, it must be considered as consequent upon some intercurrent disease.

TREATMENT.

Little need be written upon this portion of the subject. An occasional warm bath, and the application of the following unguent, is all that will be indicated.

℞ Sulphuris Sublimati,

Hydrargyri Ammoniati, - - - aa℈ij

Zinci Oxidi, - - - - - - - ʒj

Olei Menthæ Piperitæ, - - - gtt.x

Lanolini, - - - - - - - ℥j

M. Ft. ungt.

Sig· Apply two or three times daily.

In terminating this article I will be excused for indulging an expression of regret, that a very sick child prevented me devoting the time to its preparation needed to have utilized more satisfactorily the material presented.

The disease may prevail to a greater extent in Texas than I am aware of; I know of no literature upon the subject emanating from Texan Physicians. If, therefore, what I present, shall prove of interest, or lead to the recognition and study of the disease, I shall not regret the time consumed, though I may regret not writing better in regard to so interesting a subject.

In conclusion I desire to acknowledge the valuable assistance, in the preparation of this paper, rendered me by H. W. Harper, Ph. G., of Fort Worth, Texas.

Fort Worth, Texas, August 10, 1886.

## Figures and Tables

**Figure f1:**